# Method to Reduce Aerosolized Contaminant Concentration Exposure to Healthcare Workers During the COVID-19 Pandemic when Temporary Isolation Systems Are Required

**DOI:** 10.5811/westjem.2020.9.48170

**Published:** 2020-10-27

**Authors:** Bert A. Silich

**Affiliations:** Henry Ford Health System, Department of Emergency Medicine, Sterling Heights, Michigan

## Abstract

The COVID-19 pandemic has strained the healthcare system. It has led to the use of temporary isolation systems and less-then-optimum patient placement configurations because of inadequate number of isolation rooms, both of which can compromise provider safety. Three key elements require special attention to reduce the maximum and average aerosolized contaminant concentration exposure to a healthcare worker in any isolation system: flow rate; air changes per hour; and patient placement. This is important because concentration exposures of aerosolized contaminants to healthcare workers in hospitals using temporary isolation systems can reach levels 21–30 times greater than a properly engineered negative pressure isolation room. A working knowledge of these three elements can help create a safer environment for healthcare workers when isolation rooms are not available.

## BACKGROUND

Controlling both the droplet component and the aerosol component of an infectious process is critical to stopping the spread of an infection. Droplets can generally be controlled by a barrier be it gloves, masks, gowns, goggles and splash shields, tents, or isolation (intubation) boxes. The interior of some common barrier devices can create unsafe, contaminated air 21–30 times higher than inside a standard negative pressure isolation room (NPIR) when an aerosol is present. For example, the isolation box presented in Canelli et al^1^ presents an effective method for reducing the droplet component of an infection. If an aerosol component is present, it can be determined that the air contaminant concentration level inside this device will reach 21 times that of a standard NPIR in six minutes and its steady state value of 30 times that of a standard NPIR in 23 minutes.

Healthcare providers need to be aware of two potentially dangerous situations when using temporary isolation devices by considering not only the role of the droplet component but also the role the aerosol component plays in the potential to spread infections. First, consider if prior to intubation, a provider needed to attend to a patient inside a portable isolation box to access their central line for example. Assuming the provider is not wearing a powered air purifying respirator (PAPR), if their face is near or inside the opening of the isolation device their N-95 mask now has to filter air 21–30 times more contaminated then when wearing the N-95 mask in a standard NPIR. Therefore, the inhaled contaminants are 21–30 times greater than when the patient is in a NPIR without an isolation box. Second, when the isolation box is removed after intubation there is a release of air 21–30 times more contaminated than that of a patient in a NPIR without an isolation box into the local environment exposing nearby healthcare providers to these higher contamination levels.^2^ Furthermore, the use of an isolation box in a hallway could expose this highly contaminated air to other patients or visitors in the hallway.

Understanding the information and analysis presented in this paper will give healthcare providers the basic knowledge required to calculate the maximum exposure of an isolation system compared to a standard NPIR. It will also give the necessary skills to determine configuration options for patients that will minimize a healthcare worker’s average exposure to contaminants from overflow patients waiting for placement into an appropriate NPIR. This should be shared with your building engineers to determine how to minimize the concentration of contaminated air outside of the standard NPIR. This analysis only applies to an aerosolized component of contamination and does not include the effect of the droplet component, which can be reduced by local barriers.

## ANALYSIS

A reference volume, V_ref_, could refer to a room, isolation box, or even a protective hood. The ratio of the contaminant concentration in any reference volume compared to the contaminant concentration of a source, i.e., patient’s exhaled breath, is the contaminant concentration ratio (CCR).

(1) $$\text{CCR}(\text{t})=\frac{\left[\text{C}(\text{t})\text{V ref}\right]}{\left[{\text{C}}_{\text{breath}}\right]}=\left(\frac{\text{n}*{\text{q}}_{\text{breath}}}{{\text{Q}}_{\text{out}}}\right)*(1-{\text{e}}^{-\text{ACH}*\text{t}})$$

The appendix shows the derivation of this equation and other equations presented. The definition of terms is in Table 1. Equation (1) holds true if the contaminated source were placed in a negative, positive or equal pressure room because each type of pressure differential room can create the same Q_out_ (Q = flow rate) and air changes per hour (ACH) values. We know it makes sense to place a contaminated source patient in a NPIR because it helps keep those outside of this room safe.

The basic assumption is that the contaminant is fully aerosolized and mixes evenly throughout the reference volume, V_ref_. The volume flow rate leaving the reference volume, Q_out_, is typically controlled by a high-efficiency particulate air filtration system to create the desired ACH. The volume flow rate of a single (n = 1) patient’s contaminated breath is determined from the patient’s tidal volume and respiratory rate.

(2) $${\text{q}}_{\text{breath}}=\text{TV}*\text{RR }({\text{m}}^{3}/\text{hour})$$

**Figure 1:**
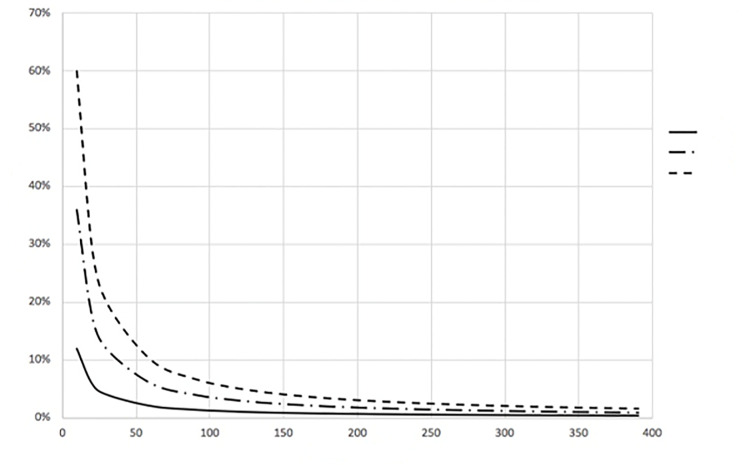
Steady state contaminant concentration ratio for various number of contaminated patients (n) for any reference volume where q breath = 1.2 m^3^/hour. *CCR*, contaminant concentration ratio.

Because the exponential portion of Equation (1) approaches zero as time (t) progresses, the CCR approaches a steady state value given by Equation (3) and is shown in Figure 1.

(3) $$\text{CCR}(\infty )=\frac{\left[\text{C}{\left(\infty \right)}_{\text{V ref}}\right]}{\left[{\text{C}}_{\text{breath}}\right]}=\left(\frac{\text{n}*{\text{q}}_{\text{breath}}}{{\text{Q}}_{\text{out}}}\right)$$

**Figure 2:**
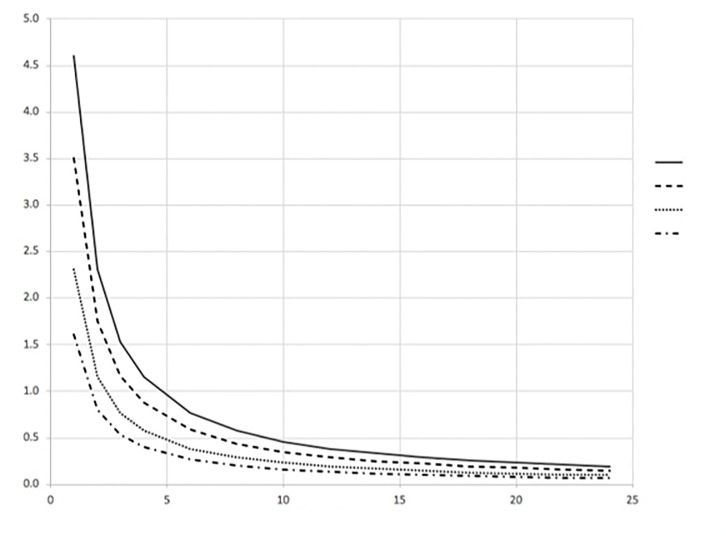
Time to reach T_ _ % of the steady state contamination concentration ratio for any reference volume. *ACH*, air change per hour.

The time to reach 99% of this steady state value (T_99%_) can also be determined from Equation (1). This result can be written as Equation (4) and is shown in Figure 2.

(4) $${\text{T}}_{99\%}=-\frac{1}{\text{ACH}}\text{ln}(1-0.99)\;(\text{hours})$$

It is vital to understand that Equation (3) tells us that the final, steady state CCR value depends on the main controllable variable Q_out_. Therefore, any two isolation systems with similar-source patients will have identical CCRs only if Q_out_ is identical in both systems. This is true even when the volumes are different. Equation (4) shows that any two different isolation systems regardless of their volumes will reach their individual steady state CCR values at the same time only if their ACH values are identical. So, Q_out_ determines the steady state CCR value and ACH determines the time to reach this steady state value.

## DISCUSSION

One goal of an isolation system is to achieve the lowest steady state CCR possible to create a safer environment for healthcare workers and other patients nearby. Equation (3) shows this is achieved by having the highest flow rate, Q_out_, possible. The CCR will be identical for any given number, n, of patients in any two isolation systems as long as Q_out_ is identical in each system. For this reason, Q_out_ is a key element to pay attention to when assessing an isolation system. Equation (4) shows the role of ACH in determining the time it takes to reach T_99%_. A larger ACH shortens this time.

A 12 ACH NPIR with a V_ref_ of 30 m^3^ has a Q_out_ of 360 m^3^/hour. Single patients are assumed to have a tidal volume (TV) of 0.5 liter and respiratory rate (RR) of 40/minute or a q_breath_ of 1.2 m^3^/hour. Therefore, this standard NPIR will reach 99% of its steady state aerosolized CCR of 0.33% in 0.4 hours (24 minutes). Simply put, the final room contaminant concentration will be 0.33% of the single patient-source contaminant concentration. The source contaminant concentration could be the patient’s exhaled breath directly, the breath exhaled after passing through a mask, or even nebulized contaminants. As previously stated, standard NPIRs require an ACH =12. For comparison ACHs for operating rooms (OR), general medicine rooms, and hospital hallways are 15, 6 and 2, respectively. An OR is kept at positive pressure while rooms and hallways are kept at equal pressure with respect to the surrounding areas.^3^

**Figure 3:**
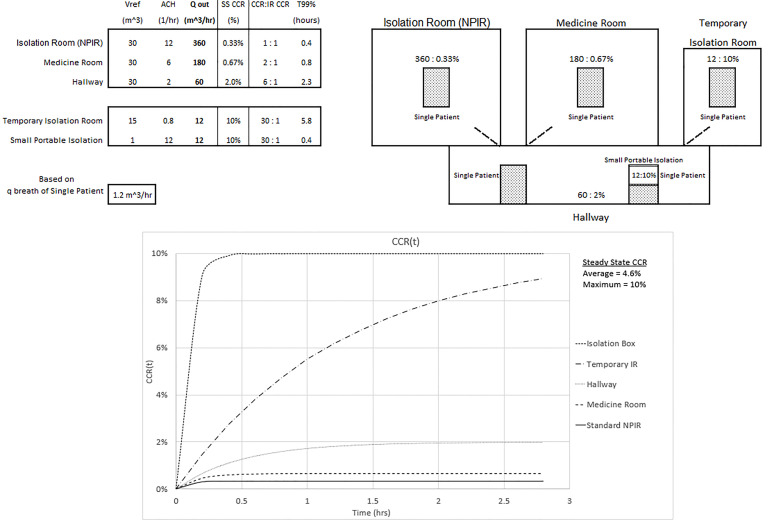
Configuration and CCR(t) of steady state average and maximum CCR exposures of 4.6% and 10% for equal time with all patients (average is 14 times the exposure of a standard isolation room CCR of 0.33%). *NPIR*, negative pressure isolation room; *SS*, steady state; *CCR*, contaminant concentration ratio.

**Figure 4:**
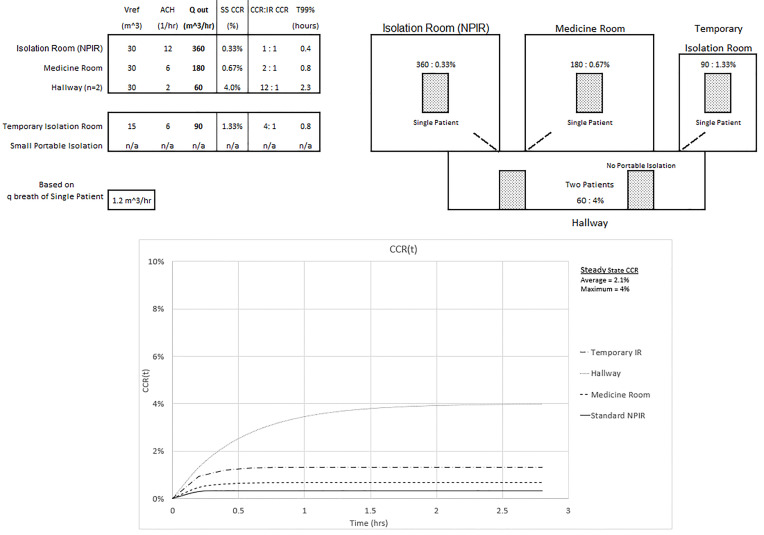
Configuration and CCR(t) of steady state average and maximum CCR exposures of 2.1% and 4% for equal time with all patients (average is 6 times the exposure of a standard isolation room CCR of 0.33%). *NPIR*, negative pressure isolation room; *SS*, steady state; *CCR*, contaminant concentration ratio.

**Figure 5:**
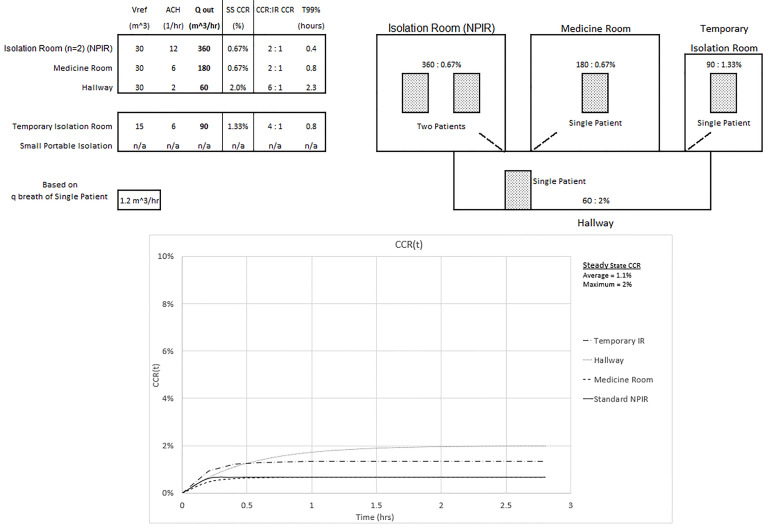
Configuration and CCR(t) of steady state average and maximum CCR exposures of 1.1% and 2% for equal time with all patients (average is three times the exposure of a standard isolation room CCR of 0.33%). *NPIR*, negative pressure isolation room; *SS*, steady state; *CCR*, contaminant concentration ratio.

The maximum and average CCR exposures for steady state conditions, assuming equal exposure time and identical patients, are given for three configurations of an overwhelmed healthcare environment without an adequate number of NPIRs (shown in Figures 3, 4, and 5). The appendix shows the average CCR at steady state for equal time with equal patients is the average of the individual CCRs at steady state. The techniques described in this paper are for emergency situations only. They are not intended to be used for an aerosolized infectious disease environment when there are a sufficient number of properly engineered NPIRs available to meet patient demand.

Figure 3 shows an overwhelmed system without a sufficient number of NPIRs where all five patients require isolation. Note the inverse relationship between the Q_out_ values and the corresponding steady state CCR. Looking at the temporary isolation room (IR) and small portable isolation devices, for example, this same relationship does not hold for the ACH values. ACH does have an inverse relationship with the T_99%_ values. The small portable system in the hallway could represent a tent or isolation box and is assumed to have a passive air exchange of 12 ACH in this setting. Realize that 360 ACH would be required to achieve a standard NPIR Q_out_ of 360 m^3^/hour. This won’t directly affect the hallway until the 10% CCR small portable container, which is not actively ventilated, is opened when a provider needs access to the patient or is removed after the patient is intubated. The temporary IR is capable of 0.8 ACH, and the CCR will also reach a 10% CCR. Twenty-four ACH would be required to achieve a standard NPIR Q_out_ of 360 m^3^/hour. The maximum CCR exposure of 10% to the healthcare worker occurs in the portable and temporary isolation systems and is 30 times the standard NPIR level. The average CCR exposure to the healthcare worker who spends equal time with each patient would be (0.33% + 0.67% + 2% + 10% + 10%)/5 = 4.6%, or 14 times the standard NPIR. These results assume each compartment’s ventilation is separate from the others. The graph of CCR(t) in the figure is obtained from Equation (1).

In Figure 4 we assume improvements were made to the ventilation system of the temporary IR that led to an improved ACH of 6 and the portable isolation system in the hallway is removed. Accounting for n = 2 in the hallway, Q_out_ still determines the CCR and ACH determines T_99%_. The maximum CCR exposure of 4% to the healthcare worker occurs in the hallway and is now 12 times the standard NPIR. The average CCR exposure to the healthcare worker who spends equal time with equal patients would be reduced to 0.33% + 0.67% + 1.33% + 2*4%)/5 = 2.1%, or six times the standard NPIR.

In Figure 5 one hallway patient is then moved into the NPIR. The maximum CCR exposure of 2% to a healthcare worker still occurs in the hallway but is only six times the NPIR standard. The average CCR exposure for equal time with equal patients is further reduced to (2*0.67% + 0.67% + 1.33% + 2%)/5 = 1.1%, or three times the standard NPIR. The tradeoff is that in the NPIR, the CCR is 0.67%, or double the “allowable” level.

Each of these three configurations offers advantages and disadvantages depending on patient diagnosis, gender, and the availability of space, equipment, and staff. These considerations are all important when deciding how to optimize patient care and healthcare safety. The patient configurations presented here demonstrate how an overwhelmed hospital environment might lead to a 3-, 6-, or even 14-fold increase in average contamination exposure to healthcare workers. Configurations different than those presented would require a separate analysis.

## SUMMARY

There are three key physical elements to understand when working with isolation systems. They are flow rate (Q_out_), air changes per hour (ACH), and patient placement, which affects the maximum and average contaminant concentration ratio exposure. Q_out_ determines the magnitude of the CCR. A larger Q_out_ will result in a smaller CCR.^4^ Matching the flow rate of any two isolation systems, regardless of their size, will give equal CCRs when the source contaminant concentrations are identical. The magnitude of the ACH determines the time the isolation system will reach 99% of its steady state value (T_99%_). A larger ACH will result in a smaller T_99%_. Matching the ACH of any two isolation systems, regardless of their size, will ensure the T_99%_ are equal in both systems. Understanding these different effects of Q_out_ and ACH are important to avoid maximum CCR exposures that can reach 21–30 times that of a standard NPIR as was shown with the small volume portable isolation box. The third key element (patient placement) becomes important when a hospital system is overwhelmed and it is not possible to place a patient requiring isolation into a standard NPIR. It then becomes important to realize that patient placement can be varied to reduce the maximum and average CCR a healthcare worker is exposed to. Based on criteria set in a specific example, it was demonstrated that optimum patient placement reduced the average CCR exposure from 14 to only 3 times that of a standard NPIR.

It is beyond the scope of this article to discuss details of other purposes for using these equations. It may not be obvious to the reader at this point, but these equations could be used as first order calculations to determine basic thresholds of ventilation required to maintain a specified safe level of contaminant concentration of aerosols in hospitals, schools, places of worship, theaters, government buildings and the like. This article should be shared with your engineering department to improve collaboration and maximize their task of optimizing ventilation to minimize exposure to infectious particles in the care of COVID-19 patients.

## Supplementary Information



## Figures and Tables

**Table 1:** Definitions of terms used to measure air contamination caused by aerosolized components.

ACH – Air changes/hour
[C] – Concentration of contaminant (particles/m^3^)
CCR – Contaminant concentration ratio
O_2_ – Oxygen supply to patient
n – number of patients in V_ref_
P – # Contaminant particles
Q, q – Flow rate (m^3^/hour)
Q_out_= ACH * V_ref_
RR – Respiratory rate (1/hour)
t – time (hours)
TV – Tidal volume (m^3^)
V_ref_ – Reference volume (m^3^)
(e.g., isolation box or room)
