# Supplementary material for: Method to Reduce Aerosolized Contaminant Concentration Exposure to Healthcare Workers During the COVID-19 Pandemic when Temporary Isolation Systems Are Required
Source: West J Emerg Med. 2020 Oct 27;21(6):93–8. doi: 10.5811/westjem.2020.9.48170 (PMC7673902; doi:10.5811/westjem.2020.9.48170)
Supplement: Supplementary file 1 [file wjem-21-93-s001.pdf]

Appendix **Contamination of an Isolated Volume**

Negative Pressure Isolation Design

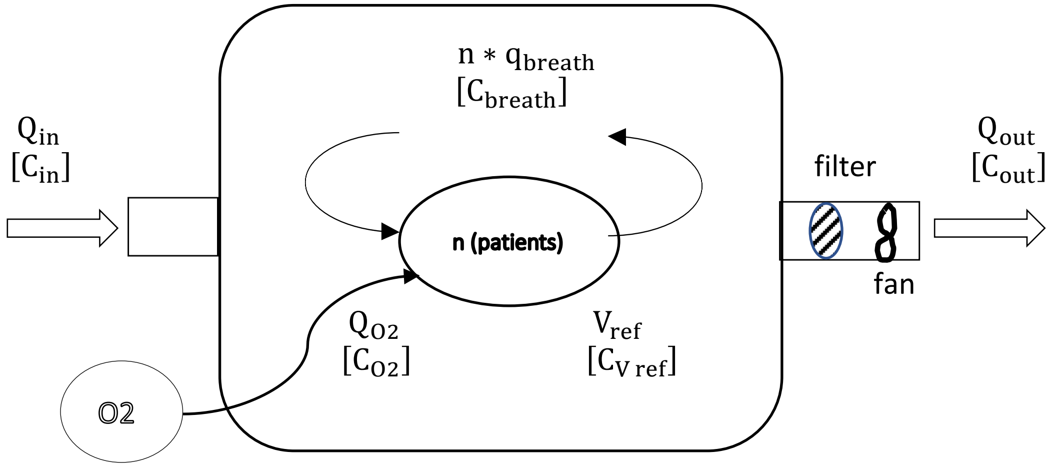

Figure 1

Definitions

ACH – Air Changes / hour

[C] – Concentration of contaminant (particles/m^3)

CCR – Contaminant Concentration Ratio

O2 – Oxygen supply to patient

n – number of patients in V_{ref}

P - # Contaminant particles

Q, q – Flow rate (m^3/hour)

RR – Respiratory rate (1/hour)

t – time (hours)

TV – Tidal volume (m^3)

V_{ref} – Reference volume (m^3) (isolation box or room for example)

Important Relationships

$[C] = \frac{P}{V}$ (particles/m^3)

$[C_{out}] = [C_{V\,ref}]$

$\overline{CCR_{average}} = \left(\sum_{i=1}^{m} CCR_i\right)/m$ for m number of equal exposure time and equal source concentrations only

$\frac{dP}{dt} = \dot{P} = Q * [C]$ (particles/hour)

flow rate of contaminant

$q_{breath} = (TV * RR)$

$Q_{out} = Q_{in} + Q_{O2}$

$Q_{out} = ACH * V_{ref}$

Solution

Assume perfect mixing of an aerosolized contaminant.

$$\dot{P}_{V\,ref} = \dot{P}_{breath} + \dot{P}_{O2} + \dot{P}_{in} - \dot{P}_{out} \quad \text{(particles/hour)}$$

Assume the patient's breath is the only source of the contaminant (e.g. virus particles), therefore $\dot{P}_{O2} = 0$ and $\dot{P}_{in} = 0$.

Appendix **Contamination of an Isolated Volume**

$$\dot{P}_{V\,ref} + \dot{P}_{out} = \dot{P}_{breath}$$

$$\dot{P}_{V\,ref} + Q_{out} * [C_{out}] = n * q_{breath} * [C_{breath}]$$

$$\dot{P}_{V\,ref} + \left(\frac{Q_{out}}{V_{ref}}\right) * P_{V\,ref} = n * q_{breath} * [C_{breath}]$$

The general solution to this 1st order, linear, ordinary differential equation is the sum of its particular and homogeneous solutions.

$$P(t)_{V\,ref} = \left(\frac{V_{ref}}{Q_{out}}\right) * n * q_{breath} * [C_{breath}] + \text{Constant}_H * e^{-\frac{Q_{out}}{V_{ref}} * t}$$

Assume the reference volume is initially clean, therefore $P(0)_{V\,ref} = 0$.

$$\text{Constant}_H = -\left(\frac{V_{ref}}{Q_{out}}\right) * n * q_{breath} * [C_{breath}]$$

The solution reveals the number of contaminant particles in the reference volume as a function of time.

$$P(t)_{V\,ref} = \left(\frac{V_{ref}}{Q_{out}}\right) * n * q_{breath} * [C_{breath}] * \left(1 - e^{-\frac{Q_{out}}{V_{ref}} * t}\right)$$

This solution can also be written as the ratio of contaminant concentration in the reference volume compared to the contaminant concentration of the source which is the patient's breath.

$$CCR(t) = \frac{[C(t)_{V\,ref}]}{[C_{breath}]} = \left(\frac{n * q_{breath}}{Q_{out}}\right) * \left(1 - e^{-\frac{Q_{out}}{V_{ref}} * t}\right)$$

Next, substitute ACH into the equation for an alternative expression of the relationship between the contaminant concentration of the reference volume to the contaminant concentration of the source.

$$CCR(t) = \frac{[C(t)_{V\,ref}]}{[C_{breath}]} = \left(\frac{n * q_{breath}}{ACH * V_{ref}}\right) * (1 - e^{-ACH * t})$$

Appendix **<u>Contamination of an Isolated Volume</u>**

It is worth noting that these results were presented using a negative pressure designed volume. Realize the resulting equations would be identical if the contaminated source were placed in a negative, positive or equal pressure room because each type of room can have the same Q_{out} and ACH. A healthcare worker can be kept safe within each of these designs. We know it makes sense to place a contaminated source patient in a negative pressure isolation room (NPIR) because it helps keep those outside of this room safe.

How to Calculate $T_{99\%}$

$$CCR(t) = \left(\frac{n * q_{breath}}{Q_{out}}\right) * (1 - e^{-ACH * t})$$

$$\frac{CCR(T_{99\%})}{\left(\frac{n * q_{breath}}{Q_{out}}\right)} = (1 - e^{-ACH * T_{99\%}}) = 0.99$$

$$(1 - e^{-ACH * T_{99\%}}) = 0.99$$

$$\ln(e^{-ACH * T_{99\%}}) = \ln(1 - 0.99)$$

$$\boxed{T_{99\%} = -\frac{1}{ACH}\ln(1 - 0.99)}$$

or

$$\boxed{T_{99\%} = \frac{4.6}{ACH}}$$

Appendix **Contamination of an Isolated Volume**

Average Contaminant Particle Exposure at Steady State

$$\overline{\mathrm{P}}_{total} = \overline{[\mathrm{C}_{\mathrm{Vref}}]} * \overline{\mathrm{q}_{\mathrm{worker}}} * \mathrm{Time}_{\mathrm{total}} = \overline{\mathrm{CCR}} * \overline{[\mathrm{C}_{\mathrm{breath}}]} * \overline{\mathrm{q}_{\mathrm{worker}}} * \mathrm{Time}_{\mathrm{total}} \quad \text{(\#particles)}$$

Represents the average number of contaminant particles a person/healthcare worker is exposed to when steady state conditions are achieved.

$\overline{[\mathrm{C}_{\mathrm{Vref}}]}$ is the average contaminant concentration in the patient's room or space.

$\overline{[\mathrm{C}_{\mathrm{breath}}]}$ is the average contaminant concentration of the source patient's exhalations.

$\overline{\mathrm{q}_{\mathrm{worker}}}$ is the average breath rate of the healthcare worker.

For a single exposure to a single patient (i) the number of contaminant particles a person/healthcare worker inhales would be

$$\mathrm{P_i} = [\mathrm{C}_{\mathrm{Vref}}]_i * \mathrm{q}_{\mathrm{worker}_i} * \mathrm{t_i} = \mathrm{CCR_i} * [\mathrm{C}_{\mathrm{breath}}]_i * \mathrm{q}_{\mathrm{worker}_i} * \mathrm{t_i} \qquad (\#\mathrm{particles_i})$$

The total # of particles inhaled for n patients is the sum of the individual particles inhaled.

$$\mathrm{P_{total}} = \sum_{\mathrm{i=1}}^{\mathrm{i=n}} \mathrm{P_i} = \sum_{\mathrm{i=1}}^{\mathrm{i=n}} \mathrm{CCR_i} * [\mathrm{C}_{\mathrm{breath}}]_\mathrm{i} * \mathrm{q}_{\mathrm{worker}_\mathrm{i}} * \mathrm{t_i}$$

By definition

$$\overline{\mathrm{P}}_{total} = \mathrm{P_{total}}$$

Therefore,

$$\overline{\mathrm{CCR}} * \overline{[\mathrm{C}_{\mathrm{breath}}]} * \overline{\mathrm{q}_{\mathrm{worker}}} * \mathrm{Time}_{\mathrm{total}} = \textstyle\sum_{\mathrm{i=1}}^{\mathrm{i=n}} \mathrm{CCR_i} * [\mathrm{C}_{\mathrm{breath}}]_\mathrm{i} * \mathrm{q}_{\mathrm{worker}_\mathrm{i}} * \mathrm{t_i}$$

Appendix **Contamination of an Isolated Volume**

At this point let us say all of the patients cared for by the healthcare worker are identical and equal time is spent with each patient. Also, the breathing rate of the healthcare worker remains constant. We can now simplify this averaging equation because

$$\overline{[C_{breath}]} = [C_{breath}]_i$$

$$\overline{q_{worker}} = q_{worker_i}$$

$$Time_{total} = n * t_i$$

Our averaging equation now becomes

$$\overline{CCR} * n * t_i = \sum_{i=1}^{i=n} CCR_i * t_i$$

or

$$\overline{CCR} = \frac{\sum_{i=1}^{i=n} CCR_i}{n}$$

The average CCR or $\overline{CCR}$ is the sum of the individual CCRs divided by the total number n.

Note: Though the author is an engineer, this math has been validated by two independent engineers with backgrounds in either hospital quality control or designing hospital ventilation systems.
